# Optical fibre sensing of turbulent-frequency motions in the oceanic environment

**DOI:** 10.1038/s41598-024-70720-z

**Published:** 2024-08-31

**Authors:** Carl P. Spingys, Alberto C. Naveira Garabato, Mohammad Belal

**Affiliations:** 1 National Oceanography Centre, European Way, Southampton, UK; 2 Ocean and Earth Science, University of Southampton, Southampton, UK; 3 Department of Mathematical Sciences, University of Liverpool, Liverpool, UK; 4 Department of Physics, University of Southampton, Southampton, UK

**Keywords:** Physical oceanography, Fluid dynamics

## Abstract

Observations of turbulence in the oceanic environment are sparse, with very few cases of coherent measurements with significant spatio-temporal extent due primarily to limitations of current observational tools. Here we propose submarine cables with embedded optical fibres as a potential solution to fill this observational gap, and utilise a recent 12-h observational optical fibre data set from a fast-flowing tidal channel to demonstrate such potential. Firstly, the presence of turbulent-scale signals driven by flow-topography interaction is shown at frequencies of 1 Hz and higher. These signals are consistent with the timing of the tidal flow as recorded by a nearby conventional sensor. Secondly, we show the presence of surface gravity waves with periods of 10 s, which are tight in frequency space further offshore but leak energy into the turbulent frequency range on parts of the cable closer to shore. This is compatible with shoreward-propagating surface waves that break in shallow water. Finally, we fit a theoretical spectral structure to the observations to show that much of the collected data (i) has a spectral slope that is consistent with the turbulent inertial subrange, and (ii) has a range of spectral energy consistent with that expected from turbulence generation by bottom drag acting on the tidal flow. In combination, these results highlight the potential for optical fibre sensing of turbulence, and call for a targeted experiment to characterise the fibre’s turbulence-sensing capabilities.

## Introduction

Many climatically-important components of the ocean system are sensitive to turbulence and mixing, including deep-ocean overturning^1,2^, primary productivity^3^, and oceanic storage of heat and carbon^4,5^. This turbulence is generated by a wide range of oceanic processes, including interactions at the oceanic boundaries and through the interior facilitated by the spatial transfer of energy within internal gravity waves. At the boundaries, both surface and bottom, there are regions of significantly enhanced turbulence driven by interactions of oceanic or atmospheric flows with the boundaries, either through direct skin drag (i.e. the friction generated as a fluid flows along a physical boundary) or through form drag (i.e. the generation of pressure anomalies by bottom boundary undulations in the direction of the flow)^6^. In this work skin drag is typically applied to the interaction between the water and the outer body of the cable and bottom drag refers to the interaction between the water and sea bed, which is driven by a mixture of skin and form drag.

Oceanic turbulent processes are currently very challenging to measure in the ocean, due to their small time and space scales and the requirement to make measurements near boundaries. As a result, observations are limited to a relatively modest number of vertical profiles collected with microstructure instrumentation^7^, which require significant ship and people resource to collect, and to a few more temporally extensive (but highly spatially confined) data sets acquired with autonomous and moored platforms^8^. A crucial limitation shared by all these turbulence observations is their suffering from a trade-off between time and space, with highly spatially-resolved data sets being poorly-resolved in time, and vice versa. This trade-off severely hampers our ability to quantitatively understand the spatio-temporal patterns, underpinning physics and wider impacts of ocean turbulence.

A potential new technological approach to the measurement of oceanic turbulent processes is provided by the distributed optical fibre sensing (DOFS) of strain and temperature^9–11^ . This technology enables measurements at very high temporal (> 1000 Hz) and spatial (< 1 m) resolutions that are continuous in time and along-cable direction, and that can be operated continually for many years. DOFS has previously been applied to a range of environmental and engineering problems^12–16^, but has only recently began to be utilised by the oceanographic community. Within physical oceanography, this has included studies examining the temperature signature of internal waves sloshing up and down sloping topography, via different optical scattering processes in bespoke and legacy seabed cables to identify signals with scales up to 5 min and 10 meters^11,17^. Additionally, other works have begun to identify the potential for distributed optical fibre sensing to detect flow both through direct coupling between the cable and the ocean in a strongly tidal region^18^ and via the modification of the propagation of acoustic signals generated by surface waves^19,20^. Together, these studies highlight the applicability of DOFS to a wide range of physical oceanographic problems.

In this study, we will demonstrate the potential for optical fibre strain measurements enabled by distributed optical fibre sensing to measure turbulent-scale processes across two regimes: firstly, the generation of turbulence by the interaction of tidal flow with the ocean bed; and secondly, the breaking of surface gravity waves as they approach the shore. The attribution of these signals to turbulent processes is then supported by inspection of the slope of the temporal spectra. Finally, it is shown that the timing and magnitude of the temporal variability seen in the offshore section of the cable is consistent with an established parameterisation for the turbulence generated by bottom drag.

## Methods

**Figure 1:**
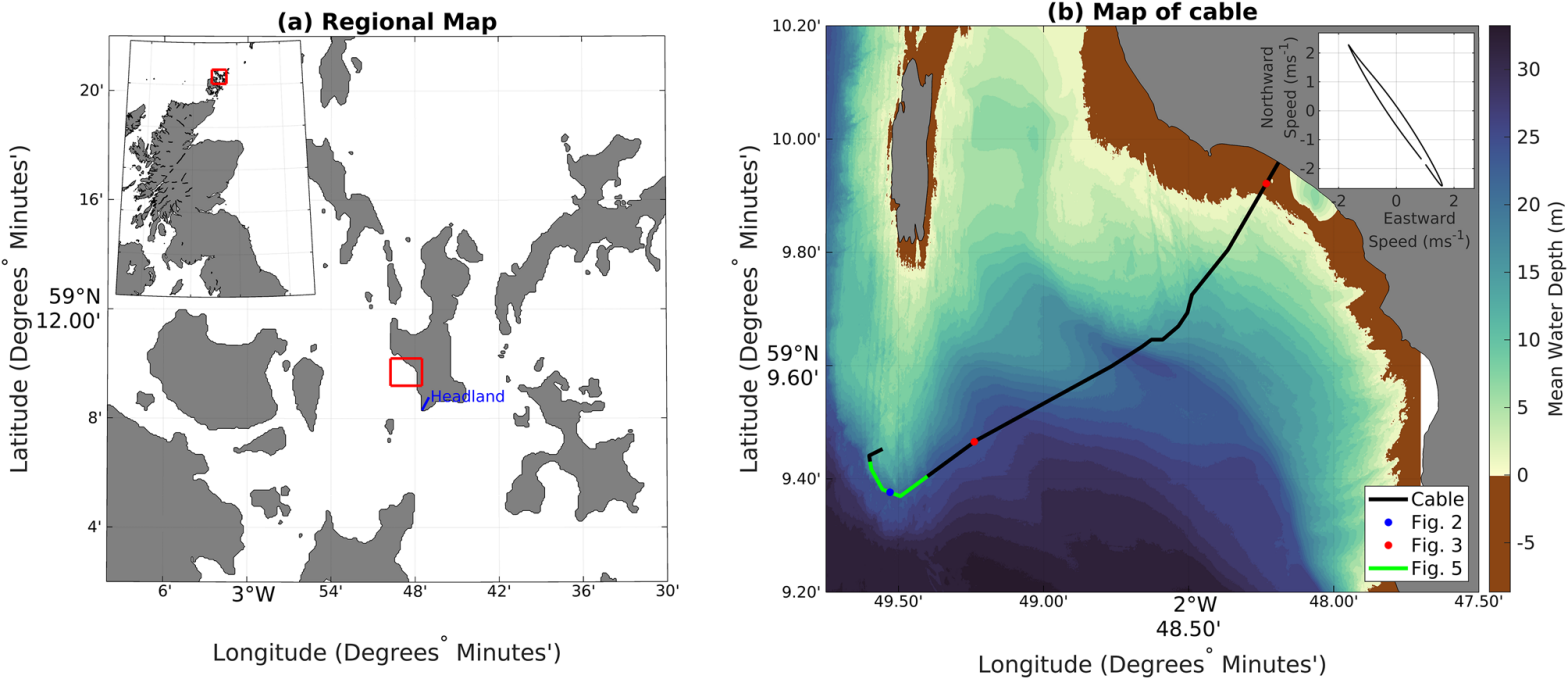
Maps showing the location of the cable. The red boxes indicate the zoomed region in the subsequent panel. The blue line in the left panel shows the location of the 1-km headland discussed below. The location of the cable is shown in blue. The locations of the wavelet transform analyses in Figs. 2 and 3 are indicated by blue and red circles, respectively. The subset of the data averaged for the lower panel of Fig. 5 is indicated by the green line. The inset is the depth-averaged velocity from the ADCP over the observational period, with axes in m s$^{-1}$. Map data from Ordnance Survey and British Geological Survey (both Open Government License).

Here we demonstrate the detection of turbulent-frequency motions with a case study, involving a 12-h interrogation of a legacy fibre optic cable at the European Marine Energy Centre grid-connected tidal test site in the Fall of Warness, off the coast of Eday, Scotland (Fig. 1). This cable is 2.1 km long and located in a channel with near-rectilinear M2 (12.42 h period) tides attaining speeds in excess of 3 m s$^{-1}$ (Fig. 1b inset). The cable is intentionally buried at the shore end but was laid exposed on the seabed through most of its length. Additionally, a 600-kHz acoustic Doppler current profiler (ADCP) was deployed 740 m to the south of the cable (59$^{\circ }$ 9.005N, 2$^{\circ }$ 49.623W) in 40 m water depth, approximately 10 m deeper than the deepest parts of the cable. The ADCP provides conventional measurements of velocity and bottom pressure every 10 min for a period overlapping with the cable observations, and so can provide oceanographic context and ground-truthing of the DOFS-observed turbulent signals.

The optical fibre was interrogated using a bespoke opto-electronic interrogation capability. The measurement comprised tracking variations in the cable properties by recording the differential changes in the phase of the back-scattered Rayleigh signal. The noise fields in the ambience of the cable physically perturb it, influencing the phase of the backscattered light that emanates due to interactions between the forward-propagating laser pulse and the fibre glass. These differential phase changes are tracked as a function of time and hence distance along the fibre, and correspondingly extracted using the optical heterodyne approach^12^. The cable is sampled using a moving-average filter with versatile along-fibre selectivity of spatial extent, often referred to as the gauge length. The evolution of a suite of measurands (e.g., pressure, temperature and strain) can be examined over the smallest possible spatial extents along the cable, by matching the spatial remit of the moving-average filter with that of the optical pulse, whilst maintaining the linearity in the recorded differential phase change^12^. In this way, a variety of oceanic phenomena affecting the cable, either through direct coupling between oceanic flows and the cable body or via pressure waves, can be derived as strain by normalising the differential phase change with the associated gauge length. The slowly-varying heating and cooling cycles of the cable’s ambient environment can also get transduced to the phase of the backscattered signal. However, in our case, the composite cable thickness (i.e. 108 mm diameter, representative of a large thermal mass) will dampen temporal variability, and cause the contributions from the thermal effect to typically manifest only at very low frequencies, if at all. As such, these contributions will be ignored in our analysis, which focuses on the 0.03 Hz - 1 Hz range and uses a 3 Hz low-pass filter, with resulting data thus likely to be dominated by directly-coupled strain effects.

For this study, the optical pulse used a 2.04 m along-cable resolution and 1000 Hz temporal repetition. These resolution choices were made bearing in mind the data storage available during the field operation, as well as the adequacy of spatial sampling needed to preserve the linear response between signals within the cable and the induced phase change in the backscattered signal. For linearity preservation between interacting vibrational/acoustic signals and the corresponding backscattered signal phase change, it is essential that the chosen gauge length be larger than the spatial resolution. Hence, a gauge length of 10 m was chosen for this study. The optical sampling frequency of the fibre optic cable sets the upper limit of the highest frequency of event that can be observed. So in this study, with a sampling frequency of 1 kHz, our upper sensing frequency is 500 Hz. It is important to note that current optical processing methods necessitate that, at any given time, there is only one optical pulse in the cable. Consequently, the upper limit to optical sampling frequency is dictated by the length of the cable.

In order to evaluate the signals detected by the cable, frequency spectra are calculated using the Fourier transform. The spectral slope is fitted to our data as $aF^{b}$, where *a* and *b* are fitted parameters and *f* is the frequency. Here we fit using a least squares approach in the frequency range 1/3 to 3 Hz at every along-cable position in 15-min time bins. This frequency range is selected as it is well within the limits of the inertial subrange set out above, but without extending beyond the 3 Hz filtering applied to the raw data. In this fitting exercise, *a* represents the magnitude of the variations in the cable time series, which we expect to be proportional to the turbulent kinetic energy dissipation rate ($\epsilon$) – with a series of unknown coefficients connected to both inertial subrange theory, the optical fibre strain methodology, and the exact coupling mechanism between the turbulent processes and the optical fibre cable. The parameter *b* is expected to be consistent with the inertial subrange predictions for the spectral slope (either − 5/3 or − 10/3, depending on the nature of the coupling) that is set out above.

## Results

Turbulent processes have a range of characteristic length scales that define them. The Ozmidov scale is the largest scale of overturning within three-dimensional turbulence, and the Kolmogorov scale represents the smallest scales at which the turbulent motions are damped by viscosity^6^. Thus, they set upper and lower bounds of turbulent flows. Previous observations in a similarly strong tidal channel showed Ozmidov scales ranging from 10 m to 100 m^21^. Given the shallow and dynamic nature of this site, we will assume that the largest overturns are set by the thickness of the water column at approximately 30 m. Taking typical values of turbulent kinetic energy dissipation in this channel of 10$^{-5}$ W kg$^{-1}$^22^ and a viscosity of 10$^{-6}$ m$^2$ s$^{-1}$ yields a Kolmogorov scale of order 1 mm. By then assuming Taylor’s frozen field^23^ (i.e. that the temporal variability of observed parameters at a fixed location is caused by the background flow, which is approximately 1-2 m s$^{-1}$, advecting turbulent features past the location), we can convert the turbulent length scales into characteristic time scales: 15-30 s and 0.5-1 ms for the Ozmidov and Kolmogorov scales, respectively.

We present two examples of turbulent-frequency motions detected by the cable-embedded fibre optic. Both examples are linked to boundary-driven processes at (1) the ocean bottom boundary, and (2) the ocean-atmosphere boundary. In both cases, the signal will be identified by performing a wavelet analysis on the cable data at specific positions. The wavelet transform converts a time series, in this case the cable strain at a specific location, into the magnitude of its frequency components. Vitally, it also allows the magnitude of these components to vary in time. This allows the simultaneous identification of the frequency content of the signal and how that frequency content varies through the 12-h observational period. In practice, here we use the generalised Morse wavelet, which provides a robust approach to estimate the presence of periodic events regardless of their shape, for example sinusoidal or ridges, and bandwidth^24^.

**Figure 2:**
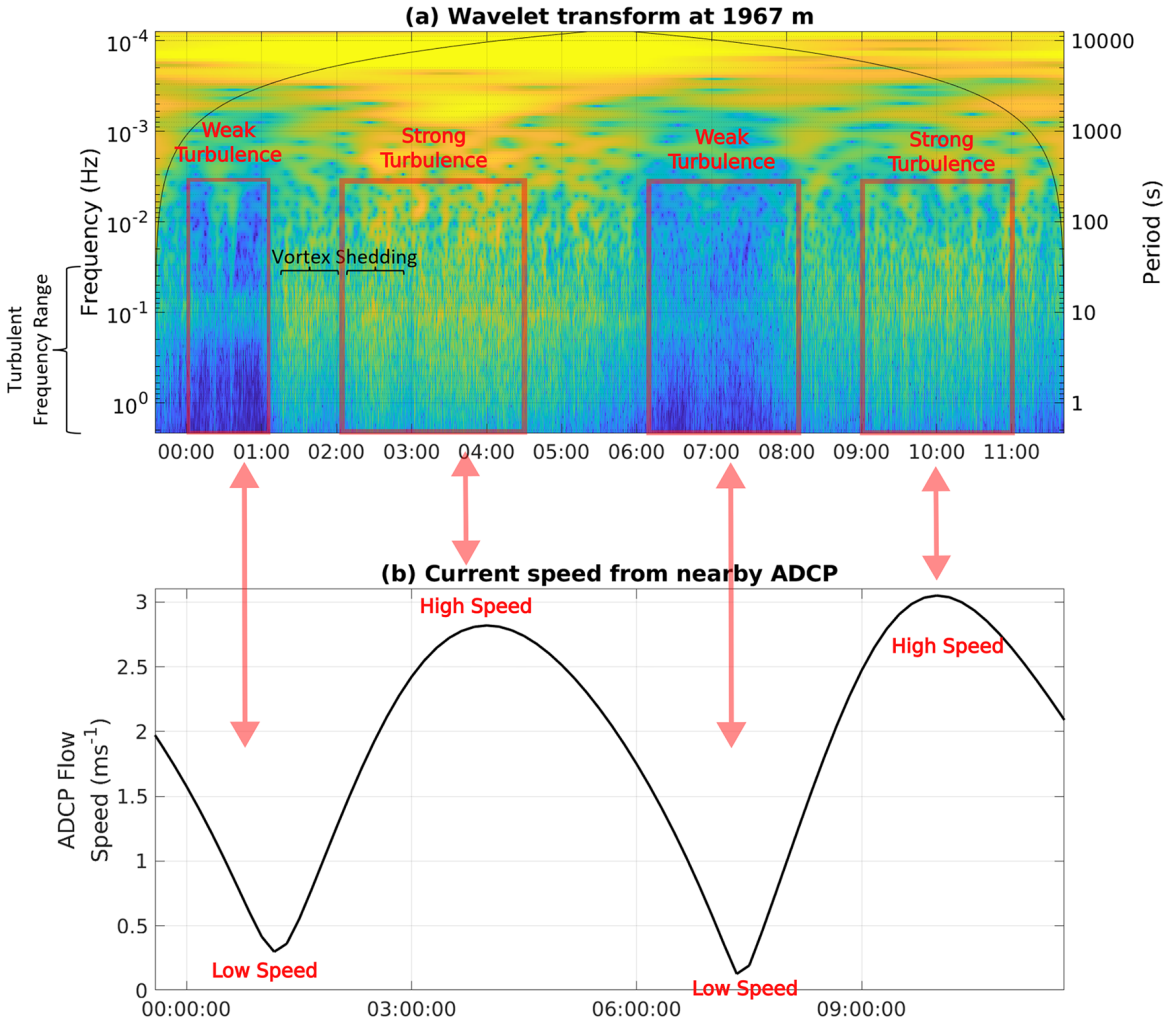
(**a**) Wavelet transform of the fibre strain at 1967 m from the onshore end of the cable. Blue indicates only a weak signal and bright yellows indicate a strong signal at that specific combination of time and frequency. The solid black lines denote the cone of influence in which edge effects become important. (**b**) Depth-averaged tidal flow speed taken from the concurrent nearby ADCP observations in 15 min time bins. Additional labels in red highlight the key features in the flow speed and turbulent scale motions.

In the first case, we consider the wavelet transform at a position at the offshore end of the cable (Fig. 2a). Here the cable is at its deepest point, and is also exposed to the fastest tidal flows within the channel. The wavelet shows the relative strength of the signal for a specific frequency at a specific time, calculated using the Morse approach. We see motions occurring simultaneously across a wide range of frequency scales, indicated by the coherence in the y direction of the variations, extending from tens of minutes, $10^{-3}$ Hz, down to less than a second, $<10^0$ Hz (the blue, e.g., from midnight to 1 am; and green, e.g., from 3 am to 4:30 am, vertical stripes in Fig. 2a). These frequencies overlap with three-dimensional turbulent scales (periods shorter than 15-30 s or $3\times 10^{-1}$ Hz) and vary coherently across all frequencies, as would be expected from turbulent processes that rapidly communicate information downscale through the inertial subrange from the large-scale (long-period) energy source towards energy dissipation at higher frequencies.

This interpretation of the frequency content of the cable data is also supported by the observed temporal modulation (variations in the x direction in Fig. 2a, b) of the turbulent-scale motions’ intensity by the tidal flow speed, as measured by the nearby ADCP. The time series of the flow speed shows a typical tidal signal with approximately 6 h cycles, with speed minima around 1:30 am and 7:30 am and maxima around 4:30 am and 10:30 am (Fig. 2b). This is consistent with the temporal modulation in the wavelet signal at frequencies less than $10^{-2}$ Hz, with a reduced signal from: 00:00 to 1:30 am; and 6:30 am to 8:00 am (the blue regions in Fig. 2a highlighted with the red boxes labelled ‘weak turbulence’) and increased energy from: 2:00 am to 4:30 am; and 9:00 am to 11:00 am (the green to yellow regions in Fig. 2a highlighted with the red boxes labelled ‘strong turbulence’). Further to this 6-h cycle, there are shorter-time scale variations that are coherent across frequency space. This includes cycles of enhanced and reduced turbulent frequency motions on time scales of approximately 40 min, most clearly visible between 1 and 3 am with regions (cycles of green and yellow around $10^-1$ Hz and higher in Fig. 2a). Taking a flow speed of 2 m s$^{-1}$ (representative of the flow magnitude seen between 1:00 and 3:00 am in Fig. 2b) and a typical Strouhal number of 0.2^25^ suggests that vortex shedding with a period of 40 min may be occurring from a feature of O(1 km) horizontal scale, comparable to the length of the headland upstream (southeast at this phase of the tide) of the cable (blue line in Fig. 1a). There are also many features present at periods of order 1 min or less, possibly associated with the formation and collapse of individual turbulent instabilities and vortices.

**Figure 3:**
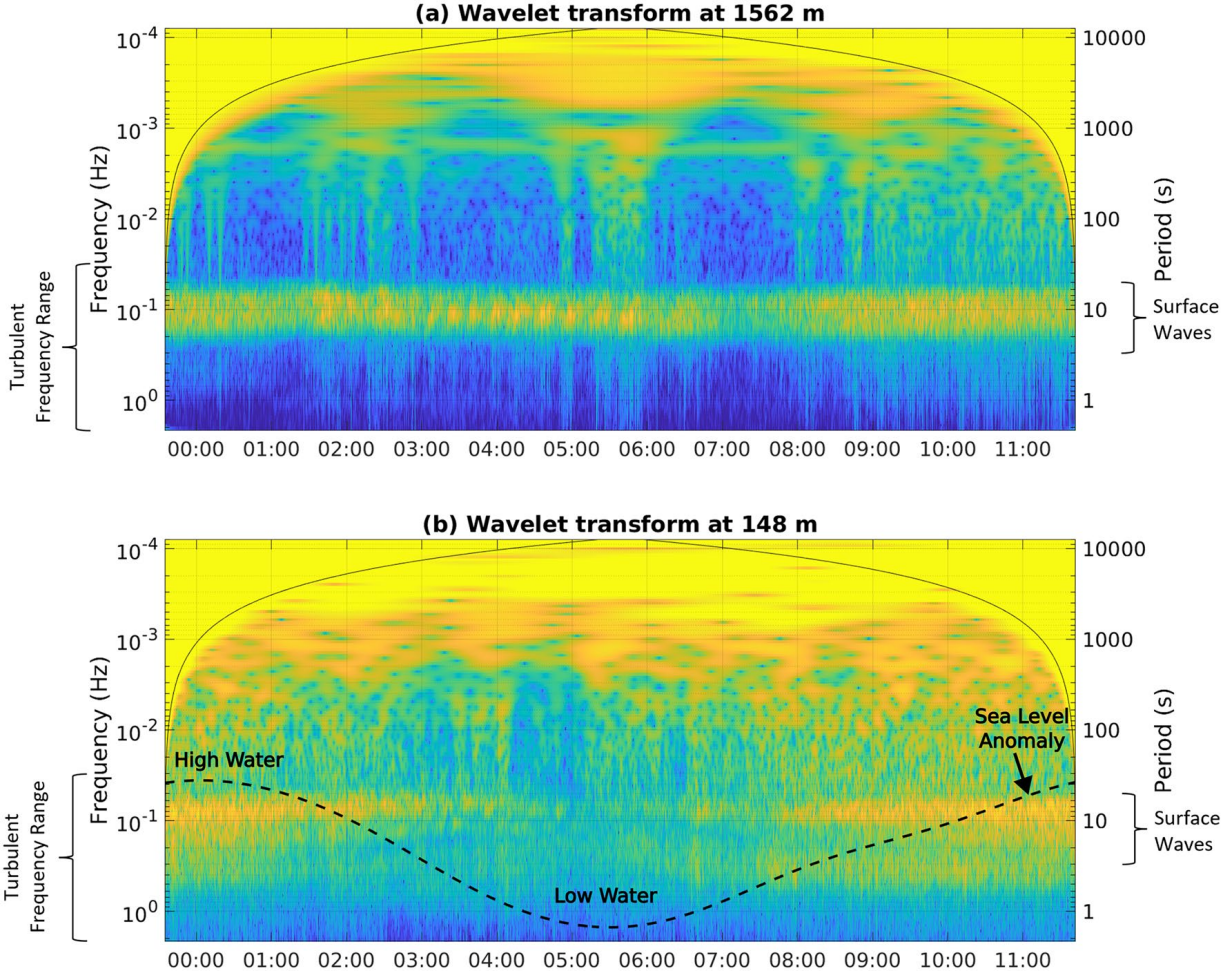
Wavelet transforms of the fibre strain at (**a**) 1562 m and (**b**) 148 m from the onshore end of the cable. Blue indicates only a weak signal and bright yellows indicate a strong signal at that specific combination of time and frequency. The solid black lines in both plots denote the cone of influence in which edge effects become important. In panel (**b**), the dashed black line indicates the sea level anomaly measured by the pressure sensor on the nearby ADCP.

Our second case demonstrates the optical fibre system’s capability to observe surface gravity waves propagating towards the shore, and the downscale impact of their breaking. A previous observational study documented surface gravity waves in this region with periods of 3 to 14 s, depending on the wind and tide regime^26^. The wavelet taken from the offshore part of the cable exhibits prominent maxima across the full time series at periods centered on 10 s and ranging from 7 to 15 s (broad green to yellow horizontal stripe in Fig. 3a around $10^{-1}$ Hz). At this offshore location, there is limited signal at higher frequencies (indicated by the blue region at frequency higher than this signal), suggesting that the surface gravity waves are stable and not transferring significant energy downscale. The wavelet transform closer to the cable’s shore end again shows a peak around 10 s (yellow peak in Fig. 3b around $10^{-1}$ Hz). However, in this occasion there is a skew toward higher-frequency motions, with the peak extending from 15 s up to 1 s, where the low-pass filter damps the signal (the coherent green signals below the yellow peak in Fig. 3b). This is consistent with the surface waves breaking as they approach the shore, and transferring their energy toward higher frequencies associated with oceanic turbulence. Again, this turbulent process is modulated by the tide in the channel, as indicated by the changes from yellow to green and then back to yellow around $10^{-1}$ Hz in Fig. 3b. Specifically, it is modulated by the tidally-induced change in water depth (the dashed black line in Fig. 3b), with this location experiencing greater surface wave and wave-breaking activity (the yellows in Fig. 3b) when the sea surface is higher (at high tide) at the start and end of the time series. This implies that the site is inshore of the main region hosting a downscale energy flux when the sea surface is low (at low tide).

Turbulent spectra can be conceptually separated into three frequency regimes with distinct physical balances: energy input at large scales / low frequencies (typically indicated by a spectral peak at the input frequency); downscale transfer of energy through the inertial subrange (typically indicated by a frequency to the power of -5/3); and dissipation of energy at small scales / high frequencies (typically indicated by a reduction to very low energy beyond the dissipation scale). The separation between the regimes is typically considered to be the Ozmidov scale for the transition from energy input to the inertial subrange, and the Kolmogorov scale for the transition to dissipation scales. Earlier, these scales were estimated as 15 s (0.06 Hz) and 1 ms (1000 Hz) for this regime. The inertial subrange has a well-defined spectral structure characterised by a progressive reduction in spectral power toward higher frequencies. For velocity or temperature, this roll off is predicted to scale as the frequency to the power of -5/3^27^. For our cable data, the coupling mechanism between the cable strain and the oceanographic turbulence is unclear. However, it may expected be related to the velocity squared if the coupling is via skin drag between the water and the outer surface of the cable^18^, which would result in a spectral slope of $(f^{-5/3})^2 = f^{-10/3}$, where *f* is the frequency. Our cable data display a clear high-frequency roll off, indicative of an inertial subrange, up to frequencies at which a flat noise spectrum becomes dominant.

**Figure 4:**
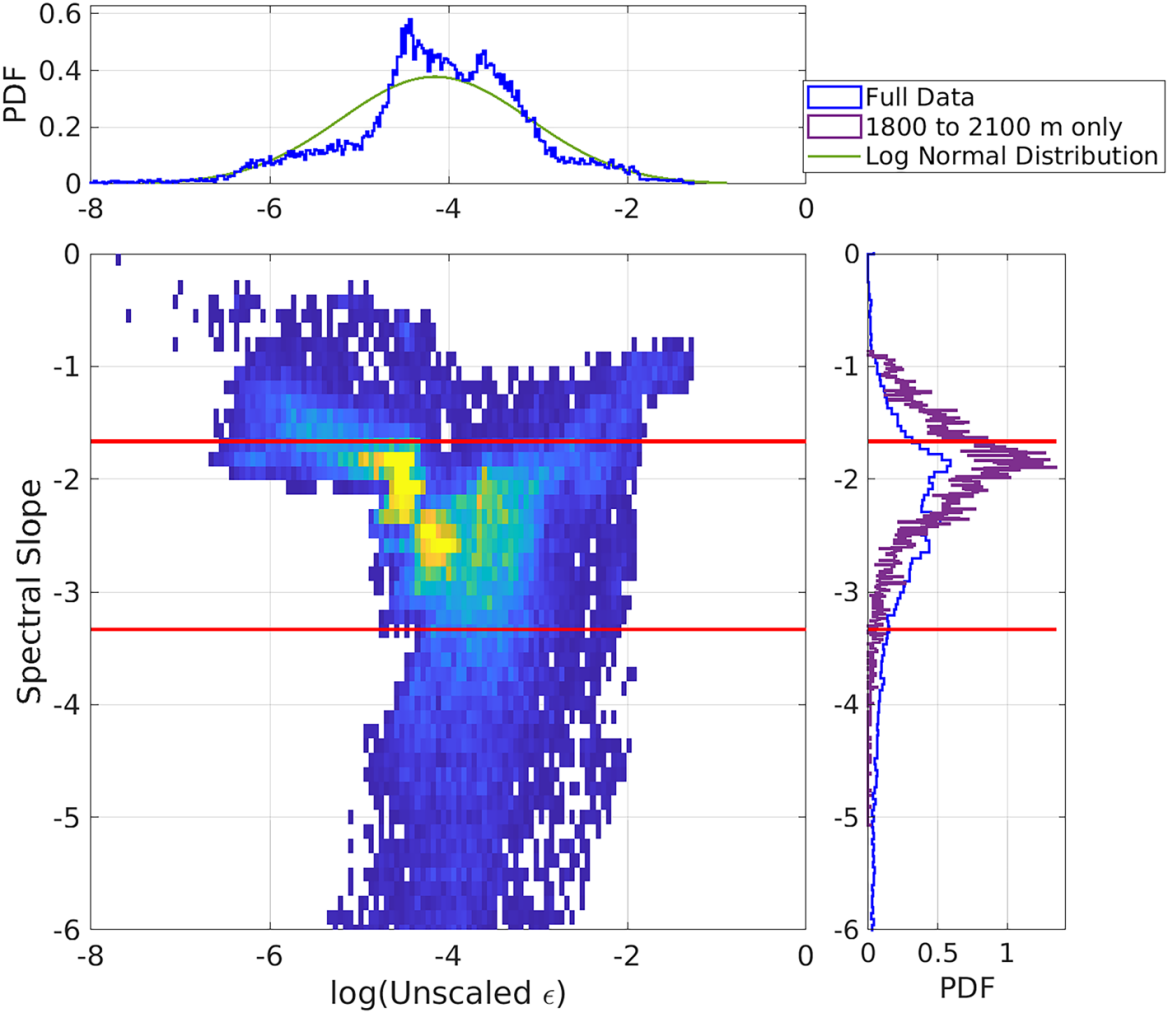
Histograms showing the parameters from the spectral fitting. In the 2-d histogram, blue indicates low count and yellow indicated high count for those intensity of turbulence and spectral slope values. Bins without data are white. Both 2-d and 1-d histograms are displayed for: (x axis and upper panel) the intensity of the turbulence; and (y axis and right panel) the spectral slope. The two red lines represent spectral slopes of − 5/3 and − 10/3. A fitted log normal distribution is also shown in green for the intensity of turbulence based on the mean, $10^{-4.15}$, and standard deviation, $10^{1.06}$, of the observations.

The fitted parameters are broadly consistent with the expectations set out above. The turbulent intensity shows a quasi-log normal distribution (Fig. 4, upper panel blue line for observations vs green line for the log normal distribution), in line with expectations based on the typical statistics of marine turbulence^28^, although it has more pronounced tails / reduced energy on the sides of the peak than anticipated. This may be due to the impingement on the cable of other intermittent processes within the frequency range examined, or to elevated background noise during low-energy data segments. Further, the peak of the spectral slope histogram is -1.86 (-5.6/3), close to expectations for direct coupling of the cable to inertial subrange turbulence, rather than being mediated through skin drag (Fig. 4, right panel). This is especially true for the most offshore section of the cable, which has a more pronounced peak in the histogram than the full data set (compare the blue for the full data set vs the purple for the offshore section only in Fig. 4 right panel). The 2-d histogram reveals that there is some relation between the spectral slope and the turbulence intensity, with weaker turbulence following the -5/3 slope indicative of direct coupling. In contrast, more intense turbulent processes tend to exhibit a steeper spectral slope, as would be expected from the turbulence’s coupling to the cable being mediated through drag. Viewed in conjunction, these results suggest that the coupling is not constant in time, and could be a function of the intensity of the turbulent process.

**Figure 5:**
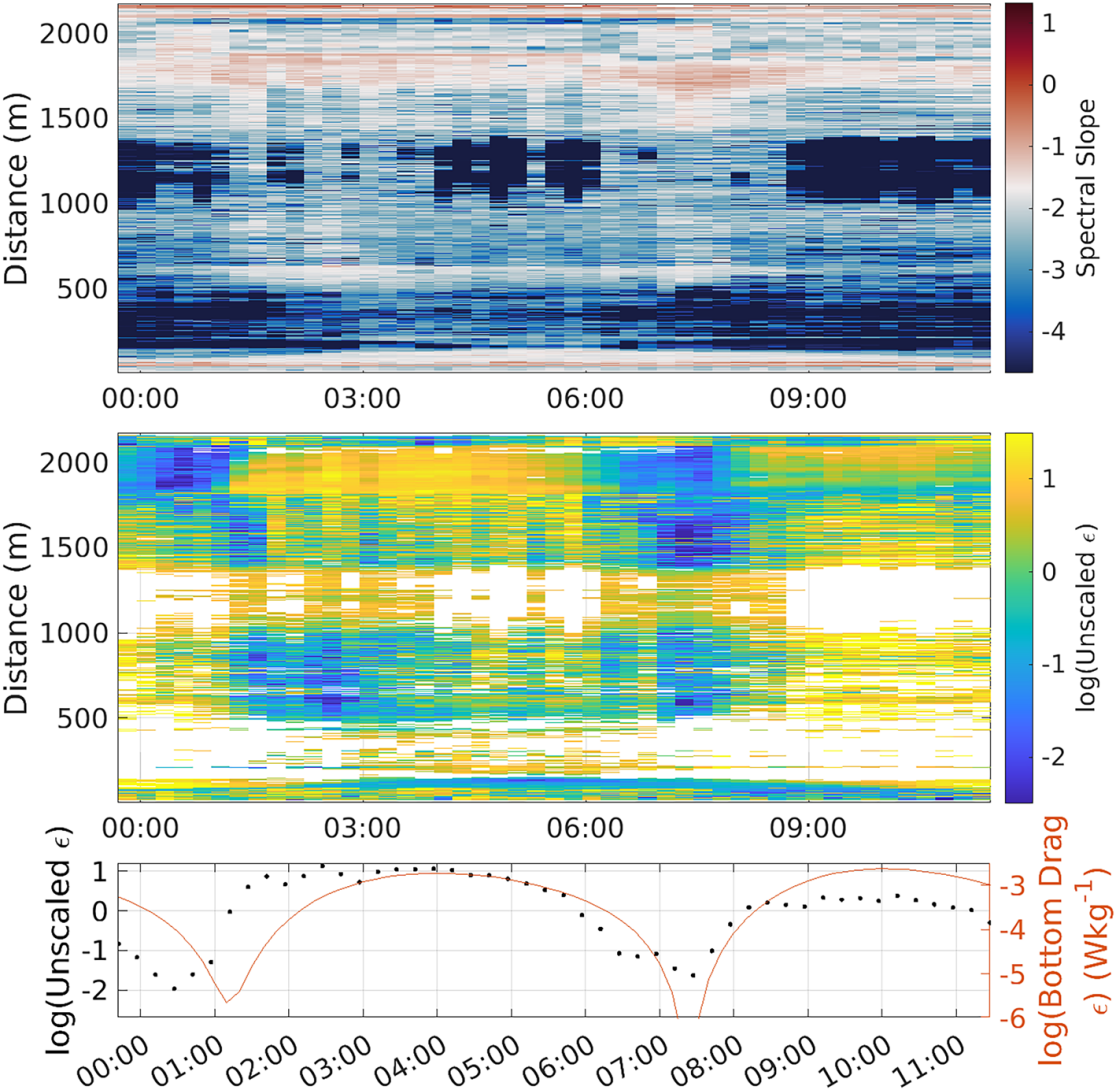
Turbulent dissipation derived from the spectral fitting approach. (upper) Hovmöller diagram of the spectral slope over the entire time series for the full cable. The white colours represent a slope of − 5/3 with reds showing a shallower slope and blues a steeper slope. (middle) Hovmöller diagram of the unscaled dissipation over the entire time series for the full cable. Regions where the fitted slope is outside of the range − 0.5/3 to 9.5/3 are excluded. In this figure, the yellows indicate more intense turbulent-scale processes, and the blues indicate weak turbulent-scale processes. (lower) Time series of the fitting-derived unscaled dissipation averaged from 1800 to 2100 m distance along the cable (black dots), and of the expected dissipation computed from the bottom drag paramterisation (red line).

There is some spatial complexity to the quality of the spectral fit, with the majority of the data being reasonably close to a slope of -5/3, but at places there are much steeper slopes occasionally exceeding -5 (Fig. 5 upper panel). These steeper slopes imply that there may be multiple processes driving signals in these frequency ranges. One potential process generating these very steep slopes could be vortex-induced vibrations (VIVs), induced by the fluid flow interacting directly with the cable. Such VIVs typically have a very well defined peak frequency, which is expected to be around 1 to 4 Hz in this location^18^. This interpretation is supported by the concurrency of periods of steep spectral slope between 1000 m and 1400 m and periods of high flow with likely more energetic VIVs. At the cable’s offshore end, where the least impact of the complex topography to the north of the cable is expected, there is a clear tidal modulation of the turbulent intensity, with two peaks over the 12-h period and weak turbulence between the peaks (blue to yellow 6-h cycle from 1800 to 2000 m distance in Fig. 5 middle panel). This modulation becomes less clear, and the turbulence more intermittent, in areas closer to shore, likely as a result of the increasing importance of complex local topography (Fig. 1) and surface wave-driven turbulence (Fig. 3). For a fast-flowing tidal channel, the interaction between the seabed and the tidal flow is expected to be the leading contributor to the generation of turbulent kinetic energy^29^. This source of turbulent kinetic energy can be estimated using a typical bottom drag parameterisation: $\epsilon = C_d |u|^3 / h$, where $C_d$ is a drag coefficient taken to be 0.0025^30^; |*u*| is the flow speed, which we obtain from the ADCP record; and *h* is the vertical scale that the energy is dissipated over, here assumed to be the full water column height of 30 m^30^. The resulting time series of bottom generation of turbulent kinetic energy is compared to the unscaled turbulent kinetic energy dissipation derived from the fitting process, averaged between 1800 and 2100 m from the shore (Fig. 5 lower panel, with the parameterised generation in red and the unscaled dissipation in black). The two time series agree well in both their temporal patterns, with co-located maxima and minima, and their approximately sinusoidal structure (Fig. 5 lower panel). Further, although the absolute values of the two records are very different due to the unknown coefficients for the conversion of the optical fibre cable data to dissipation rate, their range is similar, with both time series spanning approximately 3 orders of magnitude ($10^{1}$ to $10^{-2}$ for the unscaled dissipation and $10^{-3}$ to $10^{-6}$ W kg$^{-1}$ for the parameterised bottom drag. This supports the interpretation of the signals seen at the offshore end of the cable as being driven by turbulence generated by the interaction of the tidal flow and the bottom boundary.

## Discussion

In this work, we have documented observations of turbulent-frequency (30 s to less than 1 s) motions detected via differential strain sensing on fibre optic within a legacy seafloor energy cable in a tidal channel. These observations are interpreted in terms of two different processes: (1) turbulence generated by the interaction of tidal flow with topography; and (2) surface gravity wave breaking in shallow water. Further, we have shown that these signals have a spectral slope that is broadly consistent with classical theoretical descriptions of the inertial subrange of three-dimensional turbulence. The turbulent signals exhibit spectral energy variations of approximately the same amplitude and phase as an independent estimate of the rate of production of turbulent kinetic energy by bottom drag acting on the tidal flow. The precise relationship between the observed turbulent signals and standard measures of the intensity of turbulence (such as the turbulent kinetic energy and its dissipation rate) will be the topic of future studies. To develop this approach as a robust observational tool will require the combination of numerical simulation, lab-based experiments, and fieldwork with concurrent best-practice measurements of the turbulence. These will be needed to quantify the exact nature of the coupling between the turbulent processes and the cable potentially communicated through direct skin drag, modifications to the pressure field, or the generation of acoustic waves. It will also be vital to understand the stability over time, and robustness to different physical cables, of these parameters. Other key characteristics of the approach that need to be understood include: the interaction of the natural turbulence and the turbulence introduced by the cable; and the spatial extent of the cable sensitivity which is intrinsically linked to the coupling mechanism.

More broadly, DOFS technology has the potential to deliver a significant advance in our capability to measure oceanic turbulent processes, which has traditionally been hampered by the space-time extent of available observational approaches. This could utilise both the significant legacy infrastructure of communications cables, or the bespoke deployment of specialty cables. Additionally, the prospect of simultaneously exploiting the diverse phenomenology of light-matter interactions, which leads to a range of scattering regimes including Raman, Brillouin and Rayleigh^9,31,32^, presents a unique opportunity to obtain measurements with both absolute (very low frequency to background) and differential change (high frequency) attributes, in space and over time, using DOFS. The combination of these various scattering processes will allow the separation of multiple material properties of the fibre, coupling mechanisms and, potentially, physical oceanographic processes occurring on these scales. Further, combining the range of scattering regimes and signal processing schemes developed over recent years^9^ with different optical fibre orientations^33,34^ could facilitate comprehensive three-dimensional insights into the hydrodynamic processes affecting the cable.

In summary, DOFS provides a unique opportunity to enable wide-area, continuous, spatially distributed investigation of small-scale oceanic processes, such as turbulence. When applied to optical fibres in legacy seafloor cables, DOFS has much-reduced financial and carbon footprints relative to ship-based measurements. This study highlights the vast potential of this technology; however, further work is required to fully characterise and ground-truth its sensing capability.

## Data Availability

The datasets generated and analysed during this study are available from the corresponding author on request. Due to its large size, the dataset is not available on a public repository.
